# A Case of Thyrotropin-Secreting Pituitary Carcinoma With Bone Metastases

**DOI:** 10.1210/jcemcr/luaf332

**Published:** 2026-04-02

**Authors:** Tadasu Nagaoka, Kiyoshi Takagawa, Kunihiko Yokoyama, Toshinari Takamura

**Affiliations:** Department of Diabetes and Endocrinology, Public Central Hospital of Matto Ishikawa, Hakusan, Ishikawa 924-8588, Japan; Department of Pathology, Faculty of Medicine, University of Toyama, Toyama 930-0194, Japan; Department of Nuclear Medicine, Public Central Hospital of Matto Ishikawa, Hakusan, Ishikawa 924-8588, Japan; Department of Endocrinology and Metabolism, Kanazawa University Graduate School of Medical Sciences, Kanazawa, Ishikawa 920-8640, Japan

**Keywords:** thyrotropin-secreting pituitary carcinoma, pituitary cancer, pituitary tumor, bone metastases, hyperthyroidism, I-131 therapy

## Abstract

A 65-year-old man with a history of thyrotropin (TSH)-secreting pituitary adenoma presented with progressive spinal pain 7 years after diagnosis. Laboratory examinations revealed hyperthyroidism with markedly elevated TSH 28.5 mIU/mL (reference range, 0.3-5.0 mIU/mL) and a large pituitary tumor. Despite 3 transsphenoidal surgeries and long-term octreotide therapy, the tumor gradually enlarged. Radioiodine (RAI) therapy using iodine-131 (I-131; hereafter “I-131 therapy”) was administered for refractory thyrotoxicosis, after which thyroid hormone levels normalized but TSH rose dramatically (>500 mIU/mL). Subsequently, magnetic resonance imaging (MRI) revealed multiple spinal metastases. Biopsy of a vertebral lesion demonstrated chromophobic adenocarcinoma with positive TSH immunostaining, confirming metastatic TSH-secreting pituitary carcinoma. Palliative spinal irradiation and pamidronate temporarily relieved symptoms, but the patient died of pneumonia 8 years after onset. Autopsy revealed widespread bone metastases without any other primary malignancy. This case illustrates the malignant transformation of a TSH-secreting pituitary adenoma into carcinoma with distant bone metastases and suggests that I-131 therapy may have accelerated tumor progression. Early recognition of central hyperthyroidism and avoidance of I-131 therapy before pituitary-directed therapy are crucial to prevent treatment-related tumor exacerbation.

## Introduction

Pituitary carcinomas account for less than 0.2% of pituitary tumors and are defined by cerebrospinal or systemic metastases [1]. Thyrotropin (TSH)-secreting pituitary carcinomas are exceedingly rare, with only 3 prior cases reported. These tumors often evolve from macroadenomas over several years, acquiring aggressive features. Here, we describe a case of TSH-secreting pituitary carcinoma with extensive spinal metastases, highlighting the clinical course, management challenges, and possible treatment-related implications.

## Case Presentation

A 65-year-old man was diagnosed with TSH-secreting pituitary adenoma after presenting with hyperthyroidism, sweating, and palpitations. Laboratory findings included free thyroxine (FT4) 42.7 pmol/L (3.3 ng/dL; reference range, 10-25 pmol/L [0.8-2.0 ng/dL]), TSH 28.5 mIU/mL (reference range, 0.3-5.0 mIU/mL), and an elevated α-subunit/TSH ratio of 8.5. Magnetic resonance imaging (MRI) revealed a large pituitary mass.

**Figure 1.**
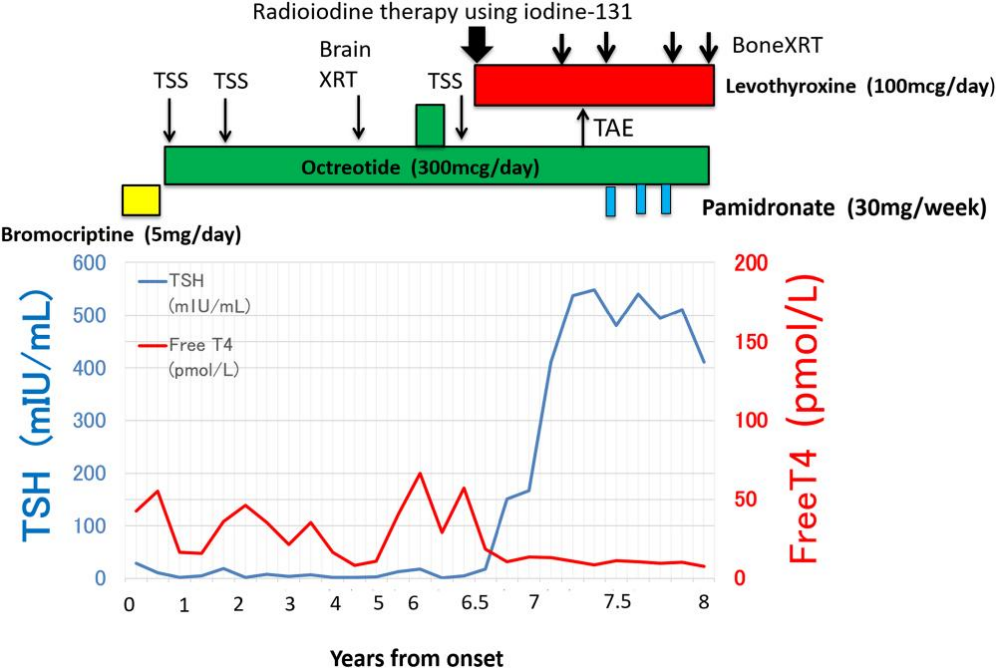
Longitudinal course of thyroid function and therapeutic interventions. The x-axis represents time (years from symptom onset). Serum thyrotropin (TSH) (mIU/mL; left y-axis, blue line with circle markers) and free thyroxine (FT4; pmol/L; right y-axis, red line with square markers) are plotted. Vertical lines indicate the timing of key treatments: transsphenoidal surgery (TSS; 3 procedures), octreotide (300 mcg/day), bromocriptine (5 mg/day), I-131 therapy, pituitary/brain external-beam radiotherapy (XRT), palliative spinal/bone XRT, transarterial embolization (TAE), levothyroxine (100 mcg/day), and pamidronate (30 mg/week). After I-131 therapy, FT4 normalized while TSH rose markedly (>500 mIU/mL), preceding recognition of widespread bone metastases at approximately 7 years.

The longitudinal course of serum TSH and FT4 levels, along with the timing of key therapeutic interventions, is illustrated in Fig. 1.

**Figure 2.**
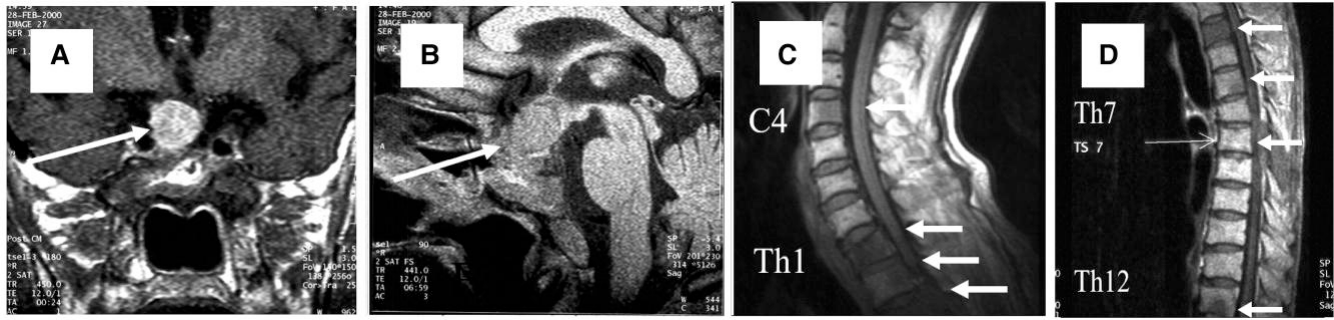
Pituitary and spinal magnetic resonance imaging (MRI) findings. A, Coronal pituitary MRI at onset. B, Sagittal pituitary MRI at onset. C, Cervical spine MRI at 7 years from onset, showing multiple bone metastases. D, Thoracic spine MRI at 7 years from onset, also demonstrating multiple bone metastases (C4, Th1–3, Th5, Th7, Th12, L2–5, and S1). Abbreviations: C, cervical; L, lumbar; S, sacral; Th, thoracic.

Representative coronal and sagittal images at presentation are shown in Fig. 2A and 2B.

Three transsphenoidal surgeries failed to control tumor growth; long-term octreotide therapy (300 mcg/day) stabilized symptoms for several years. RAI therapy with iodine-131 (I-131) was performed for refractory thyrotoxicosis. After treatment, thyroid hormone levels normalized, but TSH rose progressively (>500 mIU/mL). Seven years later, he developed right iliac pain; MRI demonstrated multiple vertebral lesions (C4, Th1-Th3, Th5, Th7, Th12, L2-L5, S1). No other malignancy was detected on systemic evaluation.

During the 7 years after disease onset, the patient was regularly monitored with MRI and pituitary hormone assays every 6 to 12 months. The pituitary tumor gradually increased in size but regressed after transsphenoidal surgery (TSS). However, following I-131 therapy, it showed rapid enlargement. No evidence of metastasis was found until the appearance of iliac pain in the seventh year (see Fig. 1).

On admission, vital signs were stable; neurological examination revealed bitemporal hemianopia and right-sided hearing loss.

Laboratory results showed alkaline phosphatase 473 U/L (reference range, 115-359 U/L), calcium 2.75 mmol/L (11.0 mg/dL; reference range, 2.10-2.60 mmol/L [8.4-10.4 mg/dL]), phosphorus 1.81 mmol/L (5.6 mg/dL; reference range, 0.81-1.45 mmol/L [2.5-4.5 mg/dL]), erythrocyte sedimentation rate 73 mm/h (reference range, <15 mm/h), neuron-specific enolase 29.0 ng/mL (reference range, <10.0 ng/mL), and persistently elevated TSH 538.9 mIU/mL with normal FT4 (10.94 pmol/L [0.85 ng/dL]) and 3,5,3′-triiodothyronine (T3) 4.04 nmol/L (260 ng/dL; reference range, 1.23-3.08 nmol/L [80-200 ng/dL]). Other pituitary hormones and parathyroid hormone (PTH)/PTH-related peptide were normal.

## Diagnostic Assessment

The diagnostic work-up included serial pituitary and spinal MRI, comprehensive pituitary hormone testing, and evaluation for other primary malignancies, as detailed in “Case Presentation.”

**Figure 3.**
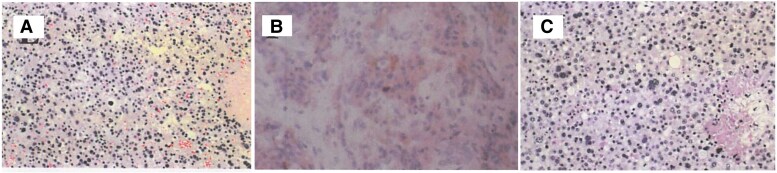
Histopathological findings. A, Hematoxylin-eosin (HE) stain of the pituitary tumor obtained at autopsy at 8 years from onset (original magnification ×20, scale bar, 2.5 mm). B, Immunohistochemical staining for thyrotropin in the metastatic bone tumor tissue at 7 years from onset, showing positive brown staining (original magnification ×20, scale bar, 2.5 mm). C, HE stain of metastatic bone tumor tissue obtained by open biopsy at 7 years from onset (original magnification ×20, scale bar, 2.5 mm).

Histopathological examination of the pituitary tumor (Fig. 3A) revealed adenoma-like cells with nuclear pleomorphism more prominent than that of typical pituitary adenomas, suggesting relatively higher-grade atypia. However, these findings alone were insufficient to confirm malignancy. In such cases, additional evaluation including mitotic count, Ki-67 (MIB-1) and p53 labeling indices, as well as clinical evidence of metastasis, is important for accurate diagnosis. In the present case, both Ki-67 (MIB-1) and p53 indices were elevated. Because histological differentiation between pituitary adenoma and carcinoma remains challenging and no standardized diagnostic criteria have yet been established, such a lesion might have been interpreted as an adenoma in the absence of metastatic findings.

The metastatic bone lesion (Fig. 3C) showed histological features similar to those of the pituitary tumor, confirming its metastatic nature. Nuclear pleomorphism was more pronounced than in the primary lesion, consistent with increased histologic atypia. Immunohistochemical staining for TSH in the metastatic tissue (Fig. 3B) was positive, confirming that the bone metastasis originated from the TSH-secreting pituitary tumor.

## Treatment

Management included multiple transsphenoidal resections, octreotide therapy, I-131 therapy, palliative spinal irradiation, internal iliac artery embolization, and pamidronate for hypercalcemia. Temozolomide chemotherapy was offered but declined by the patient.

## Outcome and Follow-up

**Figure 4.**
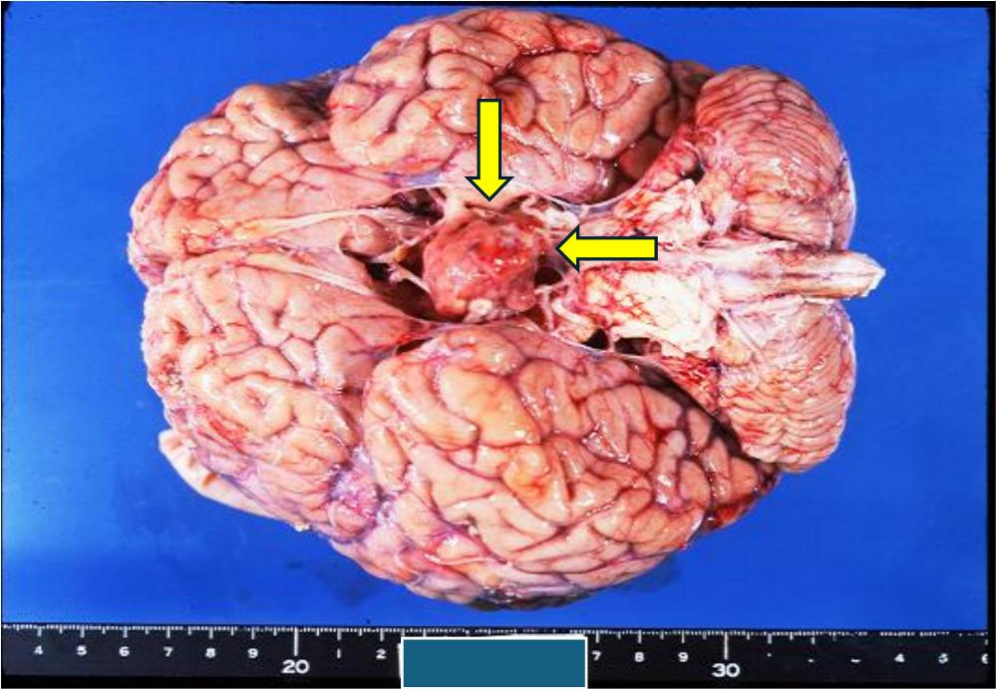
Macroscopic findings of pituitary adenocarcinoma (dimensions: 25 × 20 × 20 mm), obtained at autopsy 8 years from onset.

One year after the detection of spinal metastases, the patient developed paraplegia and died of pneumonia. Autopsy confirmed widespread bone metastases and persistent pituitary carcinoma. Macroscopic findings of pituitary adenocarcinoma (dimensions: 25 × 20 × 20 mm), obtained at autopsy 8 years from onset, are shown in Fig. 4.

## Discussion

TSH-secreting pituitary carcinomas represent an extremely rare subset of pituitary malignancies. Reported cases of TSH-secreting pituitary carcinoma, including the present case, are summarized in Table 1. These cases demonstrate variable latency periods and metastatic sites but share a tendency for aggressive behavior despite multimodal therapy.

**Table 1.** Reported cases of thyrotropin-secreting pituitary carcinoma

Author	Year	Age/Sex	Latency, y	Treatment	Metastatic site	TSH	FreeT4, SI	FreeT4 (conventional)	Reference range*^a^*
Mixson et al [2]	1993	44/M	10	Surgery, RT, chemo	Liver	180 mIU/mL	52 pmol/L	4.0 ng/dL	10.0-25.0 pmol/L (0.8-2.0 ng/dL)
Brown et al [4]	2006	36/F	7	Surgery, RT	Bone	120 mIU/mL	40 pmol/L	3.1 ng/dL	10.0-25.0 pmol/L (0.8-2.0 ng/dL)
Lee et al [3]	2012	48/F	6	Surgery, chemo	Liver, lung	95 mIU/mL	38 pmol/L	3.0 ng/dL	10.0-25.0 pmol/L (0.8-2.0 ng/dL)
Present case	2025	65/M	7	Surgery, RAI, RT	Bone (multiple)	28.5 mIU/mL	42.7 pmol/L	3.3 ng/dL	10.0-25.0 pmol/L (0.8-2.0 ng/dL)

Abbreviations: Chemo, chemotherapy; F, female; free T4, free thyroxine; M, male; RAI, radioiodine; RT, radiotherapy; TSH, thyrotropin.

^*a*^Reference ranges: TSH, 0.3 to 5.0 mIU/mL; free T4, 10.0 to 25.0 pmol/L (0.8-2.0 ng/dL).

Prior reports [2-4] indicate a stepwise progression from adenoma to carcinoma, with a latency of 3 to 10 years; our case followed a similar course over 7 years. The role of I-131 therapy in promoting malignant transformation remains speculative but has been noted in previous cases.

Although octreotide can stabilize tumor growth and normalize hormone secretion, it was insufficient in our patient after RAI treatment. Temozolomide has shown efficacy in some aggressive pituitary tumors, but patient refusal precluded its use here. Histopathologically, pituitary carcinomas lack pathognomonic features. Immunohistochemistry for Ki-67 and p53 may aid in grading aggressiveness, but variability exists. Notably, our case retained TSH immunopositivity despite advanced disease, in contrast to some reports suggesting loss of hormone staining with dedifferentiation.

Mechanistically, ablation of the thyroid with I-131 therapy reduces circulating FT4 and T3, thereby releasing negative feedback on the hypothalamic-pituitary-thyroid axis and increasing hypothalamic thyrotropin-releasing hormone drive [5]. Because TSH-secreting tumors exhibit relative resistance to thyroid hormone feedback, this enhanced drive typically further elevates tumor-derived TSH and, in some cases, may promote tumor growth or regrowth [5]. Clinical reports have described biochemical worsening and disease progression when thyroid ablation is performed before recognition of the pituitary source [6]. After I-131 therapy, levothyroxine replacement should be titrated to maintain appropriate FT4 and T3 levels rather than to normalize TSH [5]. In practice, when a thyrotropin-secreting pituitary adenoma (TSHoma) is suspected, thyroid ablation should be avoided in favor of pituitary-directed therapy (surgery and/or somatostatin analogues). If ablation has already been performed, close MRI surveillance and early initiation of somatostatin analogue therapy are advisable [5, 6]. However, enlargement of the TSH-secreting tumor and elevation of TSH levels due to the intrinsic malignant potential of the tumor itself cannot be completely ruled out.

## Learning Points

- TSH-secreting pituitary carcinomas are exceedingly rare but may evolve from invasive adenomas over several years.
- Extracranial metastases, especially to bone, are a defining feature of pituitary carcinoma.
- The potential role of I-131 therapy in malignant transformation requires further study.
- Markedly elevated serum TSH and FT4 levels at presentation may suggest aggressive biological behavior in TSH-secreting pituitary tumors.
- Early detection and aggressive multimodal therapy are crucial for management.

## Data Availability

Restrictions apply to the availability of some or all data generated or analyzed during this study to preserve patient confidentiality or because they were used under license. The corresponding author will on request detail the restrictions and any conditions under which access to some data may be provided.

## Abbreviations

free T4: free thyroxine
I-131: iodine-131
MRI: magnetic resonance imaging
PTH: parathyroid hormone
RAI: radioiodine
T3: 3,5,3′-triiodothyronine
TSH: thyrotropin
TSS: transsphenoidal surgery
